# Longitudinal MRI study over 20 years of cervical posterior extensor muscle area in asymptomatic subjects

**DOI:** 10.1038/s41598-025-08055-6

**Published:** 2025-07-01

**Authors:** Hiroyuki Izumida, Kenshi Daimon, Hitoshi Umezawa, Takehiro Michikawa, Hirokazu Fujiwara, Eijiro Okada, Kenya Nojiri, Hiroyuki Katoh, Kentaro Shimizu, Hiroko Ishihama, Masaya Nakamura, Morio Matsumoto, Kota Watanabe

**Affiliations:** 1 Department of Orthopedic Surgery, Keio University School of Medicine, 35 Shinanomachi, Shinjuku-ku, Tokyo, Japan; 2 Department of Environmental and Occupational Health, School of Medicine, Toho University, 5-21-16 Omorinishi, Ota-ku, Tokyo, Japan; 3 Department of Diagnostic Radiology, Keio University School of Medicine, 35 Shinanomachi, Shinjuku-ku, Tokyo, Japan; 4 Department of Orthopedic Surgery, Isehara Kyodo Hospital, 345 Tanaka, Isehara-shi, Kanagawa, Japan; 5 Department of Orthopedic Surgery, Tokai University School of Medicine, 143 Shimokasuya, Isehara-shi, Kanagawa, Japan; 6 Department of Orthopedic Surgery, Inagi Municipal Hospital, 1171 Omaru, Inagi-shi, Tokyo, Japan; 7 Department of Orthopedic Surgery, National Hospital Organization Saitama Hospital, 2-1 Suwa, Wako-shi, Saitama, Japan

**Keywords:** Extensor muscle of cervical spine, Longitudinal study, Magnetic resonance imaging (MRI), Asymptomatic subjects, Anatomy, Medical research

## Abstract

Few studies have investigated long-term changes in the posterior extensor muscles of the cervical spine in healthy subjects. Therefore, we used MRI to investigate changes in the posterior extensor muscles in healthy subjects over 20 years. The subjects of this study were 55 volunteers with an average follow-up period of approximately 20 years. The axial images of the C3/4, C4/5, and C5/6 levels from the initial scan and scans taken 20 years later were evaluated and compared for the following: the cross-sectional areas (CSAs) of the multifidus, semispinalis cervices, semispinalis capitis, and splenius capitis muscles, along with left–right differences, gender differences, influence of age, and muscle fatty degeneration of each muscle. The mean CSAs of the posterior extensor muscles significantly increased at C3/4 and significantly decreased at C5/6 over 20 years. The CSA of posterior cervical extensor muscles always tended to be greater on the left side than on the right side and was significantly larger in men than in women at all levels. The fatty degeneration increased significantly at all intervertebral levels. The decrease in the CSA was significantly associated with smoking status (relative risk: 2.19, 95% confidence interval: 1.32–3.63, p < 0.01), but not with clinical symptoms.

## Introduction

The biomechanics of the cervical spine muscles, its relation with cervical degenerative changes, and its association with symptoms originating from the neck has been extensively researched. Panjabi estimated that neck musculature contributes to 80% of the mechanical stability of the cervical spine^1^. Among the neck musculature, the posterior extensor muscles support the head in many situations, maintain alignment of the cervical spine, and play a part in the mobility of the neck. Consequently, dysfunction of these muscles can lead to cervical pain, malalignment, and reduced range of motion^2^. Additionally, decreased strength and endurance of the cervical extensor muscles have been reported to exacerbate cervical pain and accelerate the progression of cervical kyphosis^3–5^.

Magnetic resonance imaging (MRI) allows for the precise evaluation of the spine and surrounding soft tissues. Janssen evaluated skeletal muscles with whole-body MRI and reported that whole-body muscle atrophy progressed with age^6^. Similarly, Seo reported that atrophy of the erector spinae muscles progressed with age after the subjects reached their 40s^7^. Elliott measured cross-sectional areas (CSAs) of the cervical extensor musculature with MRI in healthy subjects to assess muscle mass at each intervertebral level^8^. Significant left–right differences in CSAs for the multifidus, semispinalis cervicis, and semispinalis capitis were observed, as well as significant vertebral level differences in CSAs of the semispinalis cervicis, semispinalis capitis, multifidus, and splenius capitis. However, this study included only females and MRIs were taken only at a single time point.

Several studies have reported longitudinal age-related changes in the cervical spine using MRI. Daimon focused on aging of the intervertebral discs in healthy subjects, while Matsumoto, Ichihara, and Watanabe reported on patients who suffered whiplash injury^9–12^. Okada studied 10-year changes in the CSAs of the posterior extensor muscles in 62 healthy subjects using MRI, and reported that increased CSAs were more common in subjects who were in their teens up to the 30 s at the initial study, whereas decreased CSAs were more common in subjects who were in their 40 s and older at the initial study^13^. The report by Okada is the only report documenting longitudinal age-related changes in the posterior extensor muscles of the cervical spine in healthy subjects, and there have been no longitudinal studies exceeding 10 years.

In this study, we evaluated the 20-year longitudinal changes in the cross-sectional areas (CSAs) and fatty degeneration of the posterior extensor muscles of the cervical spine in healthy subjects using MRI, and investigated whether these changes were associated with clinical symptoms and lifestyle habits.

## Methods

### Ethical statement

This study was approved by the ethics committee of Keio University School of Medicine (approval number 20150050), and written informed consent was obtained from all participants. Experiments were conducted following the Ministry of Education, Culture, Sports, Science, and Technology of Japan, and Ministry of Health, Labor, and Welfare of Japanese guidelines.

### Subjects

The present study, which examines the 20-year longitudinal changes of the cervical extensor musculatures in healthy subjects, was conducted as part of an ongoing longitudinal MRI study of the cervical spine^9,12^. In the initial study, whole-spine MRI images were obtained from a total of 497 asymptomatic subjects during the period from 1993 to 1996. For this study, we contacted the original subjects by mail and requested their participation in a 20-year follow-up study that was conducted between 2015 and 2017.

Among the 497 original subjects, 193 agreed to participate in the present study (follow-up rate of 38.8%), and MRI and physical examinations were conducted at eight of the original eleven participating institutions. The remaining 304 volunteers could not be followed up for the cervical MRI investigation.

**Fig. 1:**
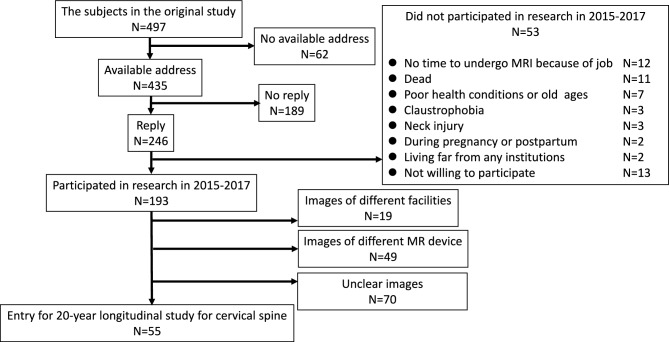
Flowchart of the subjects in the follow-up study.

The omission of required imaging sequences or the lack of image quality that allowed for clear visualization of the borders of the posterior extensor muscle led to the exclusion of 138 cases, leaving 55 cases as the subjects of this study (final follow-up rate of 11.1%) (Fig. 1). These 55 subjects comprised 13 office workers, 27 healthcare workers (doctors, nurses, and medical coworkers), 8 manual workers (construction and farming), and 7 others (housewives and retired individuals). There were 35 male and 20 female subjects, with a mean age of 31.9 ± 13.8 years at the initial study conducted 20 years ago and a mean interval of 21.9 ± 0.8 years between the MRI studies. All subjects completed questionnaires related to clinical symptoms and lifestyle habits, including smoking (regularly consumed smoking during the 20-year period) and exercise habits (regular participation in exercise for at least 1 h a week), and underwent physical examinations by spine surgeons.

The items examined by MRI included 20-year changes in the CSAs of the posterior extensor muscles of the cervical spine at the C3/4, C4/5, and C5/6 intervertebral levels, gender differences, left–right differences, age influence, and muscular fat degeneration.

This study classified the variables into three categories and employed appropriate evaluation methods to collect data for each:

- 1.Patient characteristics (age, gender).
- 2.MRI-based measurements (20-year changes in CSA, left–right CSA differences, fatty degeneration, disc degeneration, C2–C7 angle).
- 3.Lifestyle and Symptom Questionnaire (symptoms, smoking habits, physical activity habits).

We categorized age using a cutoff of 40 years at baseline, based on findings from Okada and Umezawa, who observed that individuals over 40 showed more pronounced age-related atrophy of cervical and thoracic extensor muscles. This threshold reflects a clinically meaningful point in muscle aging and has been used in prior longitudinal muscle studies^13,15^. To investigate the relationship between the change in CSA and disc degeneration, we used Matsumoto’s classification^14^ (Table 1). An increment of at least one grade in one or more intervertebral levels was regarded as a progression of disc degeneration. We also measured the C2-C7 angle on MRI sagittal images to evaluate the influence of cervical spine alignment on CSA changes over 20 years. A C2-C7 angle of less than 0° was defined as kyphosis.

**Table 1:** Grading system of cervical spine degeneration based on MRI findings.

Decrease in signal intensity of intervertebral disc	
Grade 0	As bright as or slightly less bright than cerebrospinal fluid
Grade 1	Markedly darker than cerebrospinal fluid
Grade 2	No signal
Anterior compression of dura and spinal cord	
Grade 0	No compression
Grade 1	Compression of dural sac only
Grade 2	Compression of less than one-third of spinal cord
Grade 3	Compression between one-third and two-thirds of spinal cord
Grade 4	Compression of more than two-thirds of spinal cord
Posterior disc protrusion	
Grade 0	No protrusion
Grade 1	Disc material protruding beyond the posterior margin of the vertebral body without cord compression
Grade 2	Beyond vertebral body with cord compression
Disc space narrowing	
Grade 0	100–75% of height of upper healthy disc
Grade 1	75–50% of height of upper healthy disc
Grade 2	Less than 50% of height of upper healthy disc
Foraminal stenosis	
Grade 0	No stenosis
Grade 1	Foraminal stenosis

The subjects also filled out a questionnaire regarding cervical spine-related symptoms at the time the 20-year follow-up MRI was taken. The questionnaire surveyed smoking and exercise habits, and asked for the presence and severity of neck pain, stiff shoulders, upper limb pain, and numbness of upper limbs.

### MRI examination

In the original study, a 1.5-T MRI scanner (SIGNA; GE Healthcare) was used to scan the whole spine (imaging protocol described in previous reports^14^). In the present study, images were obtained using a fast spin echo technique on a 1.5-T scanners and spine coils (SIGNA; GE Healthcare) at each institution.

**Fig. 2:**
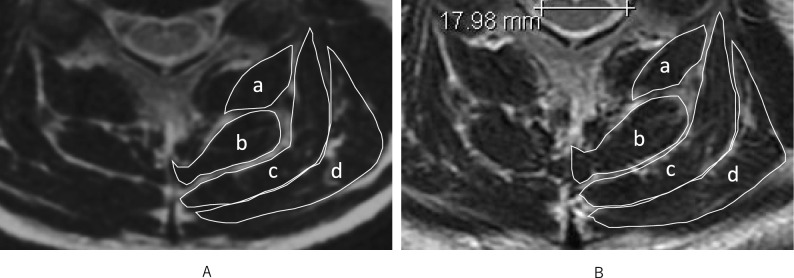
Axial T2-weighted MRI images at the C4/5 level from the same participant. (**A**) Baseline MRI. (**B**) 20-year follow-up MRI. The cross-sectional areas of the posterior extensor muscles—(**a**) Multifidus, (**b**) Semispinalis cervicis, (**c**) Semispinalis capitis, and (**d**) Splenius capitis—were segmented using ImageJ software. ROIs were manually traced within visible fascia boundaries.

The CSA of each posterior cervical extensor muscle (multifidus, semispinalis cervicis, semispinalis capitis, and splenius capitis) at the C3/4, C4/5 and C5/6 levels was measured manually using ImageJ 1.51 software. ROI boundaries were defined by manually tracing the visible fascia of each muscle on T2-weighted axial MRI slices, based on methods described by Elliott and Iizuka^8,31^ (Fig. 2). Each measurement was taken twice, and the average result was calculated.

Inter- and intra-observer errors were evaluated to measure the paraspinal muscle CSA. For the interobserver error assessment using ICC (2, 1), 20 randomly chosen MR images were measured independently by the first (H.I.) and second (H.U.) investigators. For the intra-observer error assessment using ICC (1, 2), 20 images were randomly selected, and images were measured 2 times with a 3-month interval. The values measured by the first investigator (H.I.) were used in this study.

The measurements for each muscle at each cervical intervertebral level from C3/4 to C5/6 were compared between the initial and 20-year follow-up MRI studies. CSA changes over the 20-year period were expressed as percentage change to account for inter-individual variability in baseline muscle size, following the approach used in previous longitudinal studies^13,15^. The change in muscle CSA was calculated as follows:

CSA change (%) = (CSA at follow-up − CSA at initial study) / CSA at initial study × 100

All measurements represent anatomical CSA, which can vary with body posture and does not necessarily reflect force production. This is distinct from physiological CSA, which estimates muscle force-generating capacity^31,33^.

### Evaluation of fat infiltration of the muscle

Fat infiltration was evaluated by comparing the signal intensity of a muscle’s cross-section to the signal intensity of pure muscle without fatty degeneration as reported by Umezawa^15^. Image J 1.51 was used to trim the entire posterior extensor muscle groups at the C3/C4, C4/C5 and C5/C6 levels, and the median value of signal intensity was measured. The average value of the left and right median values divided by the median value of muscle components deemed to be pure was defined as the fatty degeneration value. Each measurement was taken twice, and the average result was calculated. Fatty degeneration rate was calculated as follows:

Fat degeneration rate (%) = signal intensity in CSA of posterior extensor muscle / signal intensity in pure muscle × 100

### Statistical analysis

We used Wilcoxon signed rank sum tests for 20-year changes and Mann–Whitney U tests for comparison between CSAs. To explore the factors associated with a decrease in CSAs (CSAs change during the 20-year < 100%) after adjusted for some covariates, we applied a Poisson regression model to estimate the risk of the decreased CSAs directly. Also, we explored the association between clinical symptoms (neck pain, stiff shoulder, numbness in the upper limbs) and CSA changes. Statistical analyses were performed JMP (Ver. 11) and STATA (Ver. 16), with p < 0.05 as a significant difference.

## Results

### 20-year change of CSA

Among the original 497 participants, 193 responded to the 20-year follow-up invitation, and 55 were ultimately included after applying image quality and protocol criteria. Notably, dropout rates were higher in older age groups at baseline: 69.6% for those in their teens, 65.0% in their 20 s, 58.8% in their 30 s, 68.2% in their 40 s, 74.3% in their 50 s, and 85.4% in those aged 60 and older. This age-related attrition may affect the generalizability of the results, particularly regarding long-term changes in older adults.

In the initial study, the mean CSAs of the posterior extensor muscle group were 1458.0 ± 418.7 mm^2^ at C3/4, 1709.4 ± 499.0 mm^2^ at C4/5, and 1652.9 ± 460.3 mm^2^ at C5/6. In the 20-year follow-up study, the mean CSAs were 1582.3 ± 492.1 mm^2^ at C3/4, 1641.4 ± 480.5 mm^2^ at C4/5, and 1570.0 ± 419.1 mm^2^ at C5/6 (Table 2). The average CSA change rate over 20 years, calculated as the percentage change from baseline in paired subjects, was 109.5% at C3/4 (indicating an increase), 97.8% at C4/5, and 95.3% at C5/6 (indicating slight decreases). These values correspond to a mean increase at upper cervical levels and a decrease at lower levels, consistent with the overall trend observed. The mean CSAs of the posterior extensor muscle group showed a significant increase at C3/4 during 20 years, while there was no significant change at C4/5, and a significant decrease at C5/6. The CSA of the multifidus and semispinalis cervices muscles remained unchanged at C3/4, but showed a significant decrease at C4/5 and C5/6. The CSA of the semispinalis capitis showed a significant increase at C3/4, but no change at C4/5 and C5/6. The CSA of the splenius capitis muscle showed a significant increase at C3/4, but remained unchanged at C4/5, and showed a significant decrease at C5/6. Regarding the intra- and inter-observer errors in image evaluation, the estimated intra-observer error was 0.92 and the inter-observer error was 0.89, denoting a good agreement between these independent findings.

**Table 2:** Cross-sectional area of posterior extensor muscles.

	Multifidus (mm^2^)	Semispinalis cervicis (mm^2^)	Semispinalis capitis (mm^2^)	Splenius capitis(mm^2^)	Total (mm^2^)
Initial investigation					
C3/4	146.4 ± 48.0	180.3 ± 51.8	621.8 ± 207.9	522.8 ± 170.7	1458.0 ± 418.7
C4/5	245.6 ± 75.6	318.6 ± 102.8	594.5 ± 176.6	550.7 ± 206.7	1709.4 ± 499.0
C5/6	239.0 ± 83.1	410.8 ± 148.0	474.8 ± 148.0	527.7 ± 167.8	1652.9 ± 460.3
20-year follow up					
C3/4	147.7 ± 47.6	178.0 ± 55.9	698.8 ± 256.5*	568.0 ± 190.3*	1582.3 ± 492.1*
C4/5	224.2 ± 65.8*	289.5 ± 80.3*	608.4 ± 215.3	519.7 ± 183.2	1641.4 ± 480.5
C5/6	215.0 ± 66.4*	356.4 ± 120.1*	465.9 ± 148.9	469.5 ± 157.6*	1507.0 ± 419.1*

Comparison of the CSAs from the initial and 20-year follow up studies.

Asterisk indicates statistical significance.

### Left–right difference of CSA

In the initial study, the CSAs of the posterior extensor muscles at the C4/5 and C5/6 levels were significantly larger on the left side than that in the right side (C4/5: right 840.8 ± 240.5 mm^2^ vs left 868.6 ± 263.6 mm^2^; C5/6: right 813.0 ± 237.0 mm^2^ vs left 839.4 ± 230.2 mm^2^). In the 20-year follow-up study, the tendency remained the same with the CSAs at the C4/5 and C5/6 levels being significantly larger on the left side than on the right side (C4/5: right 804.8 ± 239.3 mm^2^ vs left 837.5 ± 246.5 mm^2^; C5/6: right 740.4 ± 207.5 mm^2^ vs left 766.4 ± 217.2 mm^2^).

### Gender difference of CSA

In the initial study, the CSAs of the posterior extensor muscle group were larger in males than in females at all levels (C3/4: male 1566.0 ± 395.2 mm^2^ vs female 1328.4 ± 416.7 mm^2^; C4/5: male 1832.3 ± 544.7 mm^2^ vs female 1561.9 ± 400.2 mm^2^; C5/6: male 1770.8 ± 470.4 mm^2^ vs female 1511.5 ± 413.8 mm^2^). In the 20-year follow-up study, the CSAs of the posterior extensor muscle group remained larger in males than in females at all levels (C3/4: male 1746.1 ± 472.1 mm^2^ vs female 1385.7 ± 449.2 mm^2^; C4/5: male 1796.9 ± 446.1 mm^2^ vs female 1454.7 ± 461.1 mm^2^; C5/6: male 1647.0 ± 411.7 mm^2^ vs female 1339.0 ± 369.4 mm^2^).

### Change in CSA by age group

**Fig. 3:**
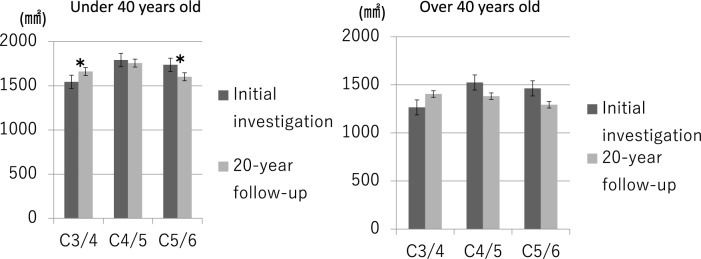
Comparison of average CSA change rates across age groups at C3/4, C4/5, and C5/6 levels. Error bars indicate standard deviations. Asterisks denote statistically significant differences (p < 0.05).

The CSAs in subjects aged under 40 years old at the initial study showed a significant increase at the C3/4 level and a significant decrease at the C5/6 level in the 20-year follow-up study (C3/4: initial study 1544.1 ± 440.3 mm^2^ vs 20-year follow-up study 1661.5 ± 508.1 mm^2^; C5/6: initial study 1737.8 ± 500.6 mm^2^ vs 20-year follow-up study 1602.5 ± 429.3 mm^2^), while the CSAs in subjects aged over 40 years old at the initial study showed no changes at any level (Fig. 3).

### Fatty degeneration

The fatty degeneration rate of the posterior extensor muscle in the initial study was 114.8 ± 8.7% at C3/4, 109.4 ± 5.2% at C4/5, and 109.2 ± 5.0% at C5/6. In the 20-year follow-up study, the fatty degeneration rates were 200.4 ± 38.6% at C3/4, 171.6 ± 29.4% at C4/5, and 169.5 ± 33.2% at C5/6, demonstrating a significant increase at all intervertebral levels. The rate of increase was more prominent at the cranial levels (Table 3).

**Table 3:** Fatty degeneration at initial investigation and 20-year follow-up study.

	Initial investigation (%)	20-year follow up (%)
C3/4	114.8 ± 8.7	200.4 ± 38.6*
C4/5	109.4 ± 5.2	171.6 ± 29.4*
C5/6	109.2 ± 5.0	169.5 ± 33.2*

Comparison of the fatty degeneration rates from the initial and 20-year follow up studies.

Asterisk indicates statistical significance.

### Factors associated decrease in CSAs

Smoking habit significantly decreased the CSAs over the 20-year period (Relative risk (RR) compared with non-smoking: 2.19, 95% confidence interval (CI): 1.32–3.63, p < 0.01). Exercise habits, progression of disc degeneration, and cervical kyphosis at initial investigation were not associated with changes in CSAs (Table 4). Furthermore, clinical symptoms was not associated with CSA changes (Table 5).

**Table 4:** Factors associated with a decrease in CSA.

		p value	Relative risk** (95% confidence interval)
Age (1-year of age increase)		0.48	1.01 (0.99–1.03)
Gender	Men	Reference	
	Women	0.70	1.11 (0.61–2.00)
Smoking habits	No	Reference	
	Yes	< 0.01*	2.19 (1.32–3.63)
Exercise habits	No	Reference	
	Yes	0.87	1.04 (0.59–1.85)
Disc degeneration	No	Reference	
	Yes	0.32	0.63 (0.26–1.55)
Kyphosis at initial investigation	No	Reference	
	Yes	0.52	1.26 (0.62–2.56)

*Statistical significance.

**Adjusted for age, gender, smoking habits, exercise habits, disc degeneration and kyphosis at initial investigation.

**Table 5:** Univariate associations between presence of clinical symptoms at follow-up and risk of CSA decrease over 20 years, assessed by Poisson regression.

	Neck pain		Shoulder stiffness		Numbness in the upper limbs	
	Relative risk (95% CI)	P value	Relative risk (95% CI)	P value	Relative risk (95% CI)	P value
Multifidus	1.03 (0.67–1.57)	0.91	0.82 (0.55–1.24)	0.35	0.86 (0.44–1.69)	0.66
Semispinalis cervicis	0.82 (0.48–1.39)	0.46	0.70 (0.44–1.12)	0.14	0.66 (0.27–1.61)	0.37
Semispinalis capitis	0.95 (0.41–2.20)	0.91	0.75 (0.35–1.63)	0.47	0.81 (0.23–2.80)	0.74
Splenius capitis	1.21 (0.77–1.92)	0.41	0.88 (0.55–1.41)	0.6	0.98 (0.49–1.95)	0.95

CSA decrease defined as < 100% of baseline. *RR* relative risk, *CI* confidence interval.

## Discussion

### 20-year change of CSA

The present study observed significant changes in the CSA of the posterior extensor muscle group over 20 years, with a notable increase at the C3/4 level and a decrease at the C5/6 level. These findings diverge from Okada’s 10-year longitudinal study, which reported consistent CSA increase at all three levels (C3/4, C4/5, C5/6), with the most pronounced increase at C3/4^13^. This difference may be due to the extended follow-up period in the present study, during which the effects of age-related muscle atrophy and progressive disc degeneration becomes more prominent.

Disc degeneration likely plays a pivotal role in the observed CSA changes. Previous research by Daimon reported that disc degeneration at the C3/4 level was lower compared to the C5/6 level across all ages groups^9^. Hui-Zi further highlighted that advanced disc degeneration was associated with reduced intervertebral mobility, particularly at levels such as C5/6^16^. These findings suggest that maintained mobility at the upper cervical spine (C3/4) preserves the function and CSA of the semispinalis capitis and the splenius capitis muscles, which are essential for head movement. In contrast, decreased mobility at the lower cervical levels (C4/5 and C5/6) likely diminishes the stabilizing role of deep muscle groups such as the multifidus and semispinalis cervicis, resulting in disuse atrophy and reduced CSA. The differential CSA changes observed across intervertebral levels underscore the interplay between anatomical function and degenerative processes. The semispinalis capitis and splenius capitis muscles, attaching to the occipital bone, are primarily involved in head movements and appear to resist atrophy when mobility is preserved. Conversely, the multifidus and semispinalis cervicis muscles, which serve a stabilizing function between vertebrae, may be more vulnerable to atrophy stemming from restricted segmental mobility and reduced biomechanical demands^17,18^.

### Left–right difference of CSA

Our study identified a constant tendency for the CSA of the posterior extensor muscle group to be larger on the left side than on the right side across all cervical intervertebral levels. Previous investigations by Elliott and Ulbrich proposed a potential association between asymmetry and handedness, but definitive evidence remains elusive^8,19^. Ichinose’s ultrasonography study of the longus colli muscle in healthy subjects revealed that the CSA of the longus colli muscle was significantly larger on the nondominant hand side compared to the dominant side^20^. This asymmetry was attributed to the role of the nondominant side in providing segmental stabilization and a feed-forward mechanism during upper limb movements of the dominant hand in daily activities or sports. Given that approximately 90% of individuals in Japan are right-handed, it is plausible that the observed left-side dominance in CSA reflects the greater stabilizing demands placed on the nondominant side. However, the absence of data on the participants’ handedness in our study limits definitive conclusions. It is worth noting that cervical musculature asymmetry may also be influenced by other factors, such as habitual posture, occupational activities, or compensatory mechanisms following minor injuries or strain.

### Change in CSA by age group

The division of subjects into under-40 and over-40 age groups at the time of the initial study, following Okada’s report^13^, provides important insights into the relationship between aging and muscle CSA. A general decreasing trend in CSA over 20 years was observed across most levels, except at C3/4. This finding is consistent with Okada’s 10-year study, which reported that individuals under 40 exhibited CSA increases, while those over 40 experienced decreases.

Age-related changes in musculature align with findings from a bioelectrical impedance analysis (BIA) conducted by Tanimoto, which indicated that trunk muscle mass increases gradually until approximately 45 years in males and 50 years in females, followed by a steady decline with age^21^. It is reasonable to hypothesize that cervical paraspinal muscles follow a similar trajectory, with functional muscle mass peaking in mid-adulthood before progressively declining. This study’s cohort, with a mean age of 31.9 ± 13.8 years at initial study, represents a demographic likely to exhibit significant muscle deterioration over the subsequent two decades.

The exception of C3/4 in the decreasing trend highlights the unique anatomical and functional role of the upper cervical musculature. The semispinalis capitis and splenius capitis muscles, which attach to the occipital bone, play a critical role in head movement and appear to maintain CSA due to sustained functional demand. In contrast, deeper stabilizing muscles like the multifidus, which are more influenced by reduced segmental mobility at lower cervical levels, are prone to disuse atrophy.

### Fatty degeneration

Fatty degeneration, characterized by the accumulation of ectopic adipocytes in muscle tissue, is a hallmark of aging. Previous research by Marcus demonstrated a consistent age-related increase in fat infiltration within thigh muscles across a wide age range (18–87 years), as assessed by MRI. Our study extends these findings to the cervical spine, revealing a significant increase in fatty degeneration across all cervical levels over a 20-year period. This suggests that the accumulation of ectopic adipocytes in cervical musculature is a progressive process influenced by aging^22^.

Interestingly, we observed that fatty degeneration was most pronounced at the C3/4 level, a finding consistent with Elliott’s MRI analysis of healthy women, which showed greater fat infiltration in the posterior cervical extensor muscles the higher the intervertebral level^23^. This pattern may reflect the functional demands and structural composition of the muscles at higher cervical levels, such as the semispinalis capitis and splenius capitis, which maintain active roles in head movement. The relatively high metabolic and mechanical demands placed on these muscles could make them more susceptible to ectopic fat accumulation over time, especially in the absence of regular, targeted exercise.

### Factors associated with changes in CSA

Our study found a significant association between smoking and decreased CSA of the posterior cervical extensor muscles. In contrast, prior studies by Okada and Umezawa, examining 10-year changes in the CSA of cervical and thoracic extensor muscles, respectively, did not identify such a relationship^13,15^. This discrepancy may stem from the longer follow-up period in the current study, during which the cumulative effects of prolonged smoking likely exacerbated muscle atrophy.

Nicotine’s impact on muscle health is well documented. It reduces insulin secretion from pancreatic beta cells, impairing muscle protein synthesis while accelerating protein degradation. Smoking also triggers the production of pro-inflammatory cytokines, such as TNF-α and IL-6, and activates NF-κB, which promotes muscle-specific RING-finger proteins involved in muscle atrophy^24,25^. Moreover, smoking-induced reductions in forced vital capacity (FVC), a marker of overall muscle health, may contribute to decreased CSA. Anong’s research demonstrated that smokers exhibited significantly lower FVC compared to nonsmokers, suggesting a systemic impact of smoking on muscle mass^26^.

Despite identifying smoking as a risk factor for reduced CSA, no associations were found between CSA and clinical symptoms such as neck pain, which is often attributed to overload or fatigue of cervical extensor muscles. Elderly individuals, prone to progressive kyphosis, often experience increased posterior cervical muscle loading due to forward head posture, which leads to chronic contraction, circulatory disturbances, and pain induced by metabolite accumulation^27,28^. However, the absence of significant kyphosis progression among our subjects may explain the lack of correlation between decreased CSA and neck pain in this study.

Okada’s research linked reductions in the CSA of the cervical semispinalis muscle to the development of stiff shoulders^13^, which Takagishi defined as a combination of muscle tension, heaviness, and dull pain extending from the neck to the scapula^29^. However, the precise mechanisms underlying stiff shoulders remain unclear, and other factors beyond CSA reduction likely contribute to their development. This complexity may explain why no significant association was identified in the present study.

### Study limitations

There are several limitations in this study that should be considered when interpreting our results. First, we measured muscle volumes only on T2-weighted axial images because axial images were acquired only with a T2-weighted pulse sequence in the initial trial protocol. Previous reports have investigated muscle structure by using T1-weighted^8,23,30,31^ or T2-weighted^32–34^ images, and the superiority of either sequence in illustrating the muscle structure remains controversial. Additionally, the baseline images were obtained from film, which introduces noise and contrast inconsistencies, limiting the use of threshold-based segmentation methods such as Otsu’s technique. As a result, we adopted a method previously described by Umezawa^15^. That compares muscle luminance to reference tissue to assess fatty infiltration. However, this technique is not yet widely validated or standardized, and may be less accurate than advanced methods such as Dixon imaging or intensity-based thresholding. We recognize this as a limitation of the study, and recommend future studies adopt validated segmentation techniques if imaging quality allows. Second, fast spin echo sequences on 1.5-T scanners were used in both studies, but the types of MR scanners and imaging parameters were not identical between the two studies, which might have brought about differences in the quality of images between the initial and second MRI 20 years later. To minimize the differences in image quality, large numbers of subjects scanned with 0.5-T scanners in the initial study and all poor-quality MR images generated by 1.5-T scanners were excluded from this study. As a result, the estimated intra- and inter-observer correlation, which was performed to assess reliability and reproducibility of measurements, showed good agreement. Third, all participants in the initial cohort were asymptomatic at baseline, and symptom data were collected again at the 20-year follow-up MRI. Therefore, any reported symptoms reflect new onset during the follow-up interval. However, due to the lack of detailed longitudinal symptom tracking, the associations between CSA and symptoms were assessed cross-sectionally at follow-up only. This limits our ability to determine temporal relationships between muscle changes and symptom development. Additionally, cervical alignment was assessed using MRIs performed in the supine position. As spinal posture is influenced by gravity and muscle tone, kyphosis observed in the supine position may underestimate postural deformity compared to upright assessments. This limitation may influence interpretation of the relationship between spinal alignment and cervical muscle degeneration. Fourth, the study included participants with various occupational backgrounds, such as office workers, healthcare professionals, manual laborers, housewives, and retirees. Differences in occupational physical demands may have influenced cervical muscle CSA changes. Due to the limited sample size, we were not able to control for employment status in the analysis, which may limit the generalizability of the findings. Furthermore, the follow-up rate was not high (11.1%), indicating the possibility of selection bias. Nonetheless, this is one of the few longitudinal studies on age related changes in the posterior extensor muscles of the cervical spine in healthy individuals. The age-related changes in the posterior extensor muscles described in this study can serve as a control for the assessment of changes in the posterior extensor muscles associated with various cervical spinal disorders and with non-surgical and surgical treatments for these disorders.

## Conclusion

This study investigated 20-year changes in the posterior extensor muscles of the cervical spine in 55 healthy subjects using MRI. The findings revealed significant anatomical and physiological changes over time. The cross-sectional area (CSA) increased significantly at the C3/4 level, remained stable at C4/5, and decreased significantly at C5/6. Additionally, fatty degeneration progressed at all levels, even in the absence of clinical symptoms or pathological conditions, underscoring its role as a natural consequence of aging. Smoking was identified as a significant factor associated with a reduction in CSA, highlighting the impact of lifestyle choices on cervical muscle health.

These results provide valuable insights into the aging process of the cervical musculature in healthy individuals. They serve as a baseline for understanding the effects of aging and lifestyle factors on cervical spine health, with potential implications for the prevention and management of cervical spine disorders. Future studies should focus on larger, more diverse populations and explore the interplay of aging, lifestyle, and therapeutic interventions on cervical spine musculature.

## Data Availability

The datasets used and analyzed in the current study can be acquired from the corresponding author upon reasonable request.
